# Cardiopulmonary exercise capacity markers and their link to symptom burden in patients at risk for heart failure with non-reduced ejection fraction

**DOI:** 10.1038/s41598-025-94172-1

**Published:** 2025-03-15

**Authors:** Stefan Kwast, Jana Hoffmann, Christoph Pökel, Roberto Falz, Antina Schulze, Thomas Schröter, Michael Andrew Borger, Martin Busse

**Affiliations:** 1 Institute of Sports Medicine, University Leipzig, Leipzig, Germany; 2 Sports Medicine Outpatient Clinic, University Leipzig, Leipzig, Germany; 3 Department of Cardiac Surgery, Leipzig Heart Center, University Leipzig, Leipzig, Germany

**Keywords:** Cardiac power output, Heart failure, Exercise performance, KCCQ, Exercise intolerance, avDO_2_, Cardiovascular diseases, Heart failure, Quality of life

## Abstract

The American Heart Association (AHA) guidelines assess heart failure (HF) via comorbidities, laboratory markers, and echocardiography, while the New York Heart Association (NYHA) classification evaluates functional capacity. The primary objective of this study was to investigate the correlation between objectified HF-related symptoms and cardiac and muscular exercise capacity in Stage B HF patients with non-reduced ejection fraction. As secondary endpoints, we stratified this analysis for subgroups of NYHA classes to evaluate the primary endpoint for different levels of impairment and for sex to address for differences between men and women. Sixty-two Stage B HF patients with non-reduced EF were screened from an HF-risk cohort. Assessments included medical history, HF-related symptoms (Kansas City Cardiomyopathy Questionnaire, KCCQ), physical examination, laboratory tests, echocardiography, and cardiopulmonary exercise testing (CPET) with cardiac output monitoring. Correlations were analyzed between KCCQ score and exercise capacity markers: maximal oxygen uptake (VO_2_max), arterio-venous oxygen difference (avDO_2_), cardiac power output (CPO), mean arterial pressure (MAP), and respiratory efficiency (Ve/VO_2_). Subgroup analyses were performed by sex and NYHA class determined by VO_2_max or KCCQ functional scores. Our HF patient cohort showed reduced KCCQ scores (78.3) and VO₂max (22.9 ml/kg/min), and a progressed reduction in avDO₂. In the total cohort, KCCQ scores showed moderate correlations with Ve/VO₂ (*r* = -0.39) and MAP (*r* = 0.27). NYHA stratification by VO₂max revealed differences in avDO₂ and cardiac output but not KCCQ scores, while KCCQ-functional stratification only showed differences in Ve/VO₂. Sex-specific analysis showed KCCQ scores correlated with CPO in men (*r* = 0.65) and Ve/VO_2_ in women (*r* = -0.68). Our identified Stage B HFpEF cohort showed already alterations in total, cardiac and muscular exercise limitation. The HF symptom severity was weakly associated to the higher blood pressure and ventilatory inefficiency and, but moderately to strongly correlated CPO in men and Ve/VO_2_ in women in sex-specific analyses.

## Introduction

Heart failure (HF) is a syndrome characterized by various symptoms, including shortness of breath, edema, and increased fatigue during daily activities^1^. In the early stages of HF, patients typically exhibit mild symptoms and functional limitations^2^. As HF progresses, chronic myocardial pressure-volume overload and reduced cardiovascular exercise capacity lead to severe health outcomes, including increased mortality risk, hospitalization, and healthcare burdens^3^.

The New York Heart Association (NYHA) classification is based on subjective patient symptoms and corresponding functional status^4^. This classification accounts for cardiac, pulmonary, and muscular performance impairments, thereby reflecting HF as a syndrome^5,6^. The current guidelines from the European Society of Cardiology (ESC) and the American Heart Association (AHA) primarily propose a heart-specific classification and staging, focusing on predisposing factors, diagnostic assessments, the presence of structural heart disease or laboratory assessments of cardiac enzymes^7,8^. So, the NYHA functional classes and ESC/AHA staging co-exist as parallel assessments.

For early stages of HF, according to AHA HF stages A and B^7^, there is limited evidence about cardiac and muscular capacity alterations and their specific contribution to functional capacity classification (NYHA class) and heart failure related symptoms. Particularly in patients with heart failure with preserved ejection fraction (HFpEF), the multifactorial genesis of cardiac overload and associated limitations in other exercise capacity, such as skeletal muscle exercise capacity, have not been studied in detail.

Hawwa et al. demonstrated a correlation between NYHA class, maximal oxygen consumption, and patient-reported outcomes assessed via the Kansas City Cardiomyopathy Questionnaire (KCCQ) in patients with chronic heart failure but did not differentiate for exact quantification of cardiac and skeletal muscle capacity^9^.

Cardiopulmonary exercise testing (CPET) can reveal objectified limitations in functional capacities in HF patients^10–12^. Reduced exercise capacity is not solely due to cardiac dysfunction^13,14^ as HF as a syndromic disease is also influenced by the reduced exercise capacity of the peripheral skeletal muscles^15^, increased afterload due to arterial hypertension or chronotropic incompetence^16^. Specifically, the left ventricular cardiac power output and stroke work determine heart functional capacity during exercise stress tests^17,18^, particularly in HF patients with altered chronotropic variability^19^.

In the early stages of the disease, it can be hypothetically assumed that there is already an advanced limitation in overall functional capacity. This exercise performance limitation could arise secondary to other diseases and frequently leads to a capacity limitation or dysregulation of peripheral muscles, as well as vascular and pulmonary systems. Recent studies highlight those comorbidities such as hypertension, diabetes, and obesity play significant roles in exacerbating these alterations^20^. Additionally, poor cardiorespiratory fitness has been linked to higher morbidity and mortality rates in HFpEF patients, underscoring the need for targeted interventions^21^.

One difficulty in the early detection of HF patients is that NYHA class patients are almost symptom free. Therefore, physiological changes at the onset of HF may not yet be associated with specific symptoms and their severity. Consequently, it is useful to consider patients separately according to NYHA class I and greater I. Gender differences have also been observed in HF patients without reduced ejection fraction^22,23^. Therefore, it is important to consider sex as a confounding factor or to analyze it separately^23^, especially in the early stages of the disease.

The purpose of this cross-sectional study was to gain a deeper understanding of the interaction between the functional NYHA classification, heart failure-specific patient-reported outcome measures (PROMs) assessed by the Kansas City Cardiomyopathy Questionnaire (KCCQ), and objectified exercise capacity markers in Stage B HF patients with non-reduced EF/preserved EF. Therefore, three main research objectives were addressed: (I) to correlate the KCCQ score with physiological cardiac, muscular and ventilatory exercise markers; (II) to asses and compare these exercise performance parameters in NYHA Class I and II patients classified by (a) maximum oxygen uptake and (b) KCCQ functional score; and (III) to analyses exercise markers and KCCQ scores stratified by sex.

## Materials and methods

This was a cross-sectional, observational study. The data reported were part of routine medical assessments at the Sports Medicine Outpatient Clinic of the University of Leipzig, prior to trial-specific interventions and randomization for the HITS-trial (Heart failure, Individualized training, Telemonitoring and Self-Management). Methods and data were parts of the protocol of a prospective, randomized, controlled trial „HITS-Trial “, approved by the Ethics Committee of the University of Leipzig (479/19-ek). The study was registered in the German Clinical Trial register DRKS00019022 (28.05.2020) and the study reporting follows the STROBE guideline for reporting of cross-sectional studies^24^.

### Subjects

Patients with a risk constellation for heart failure (AHA HF stage A)^7,8^ at the outpatient clinic for sport medicine were screened for heart failure according to the AHA and ESC guidelines. Patients with manifest comorbidities related for elevated NTproBNP levels, like chronic kidney diseases, or HF symptoms, like COPD, were excluded from the screening procedure for the observational cohort. All patients were in a stable cardiovascular clinical situation.

**Fig. 1:**
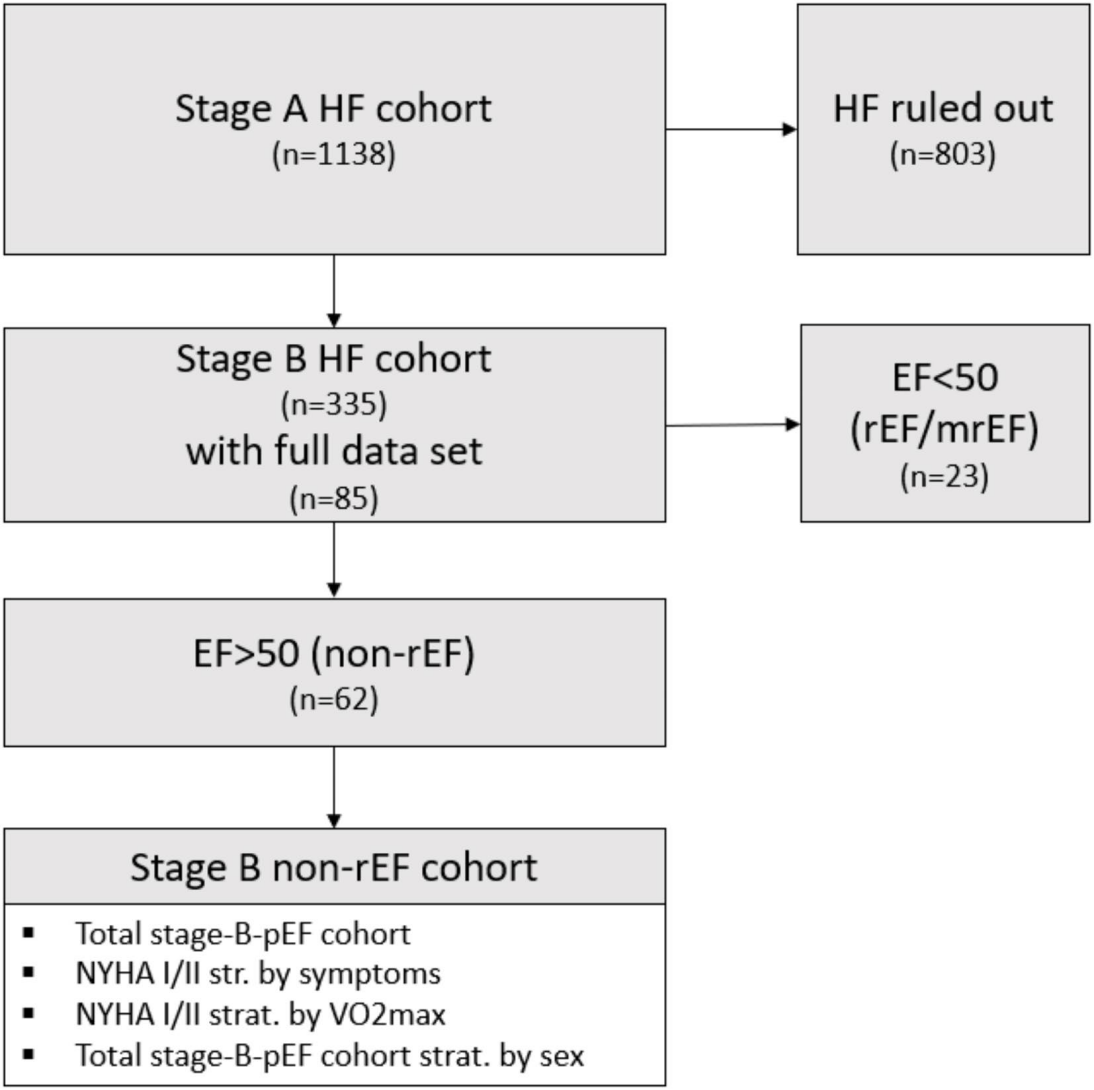
Patient identification and cohort selection; EF: ejection fraction, mrEF: moderate reduced ejection fraction; non-rEF: non-reduced ejection fraction; NYHA: New York Heart Association; pEF: preserved ejection fraction; rEF: reduced ejection fraction, HF: Heart failure; VO_2_max: maximum oxygen consumption.

A total of *n*= 62 patients met the criteria^8^ for stage B HFpEF diagnosis, including elevated NTproBNP levels, specific risk factors, comorbidities and HF-related symptoms, and had a full evaluable diagnostic data set. Not included for further analysis were 23 patients assessed with reduced EF. All participants of the identified stage B HFpEF/non-rEF were verbally informed and gave their written consent. The cohort selection process is illustrated in Fig. 1.

The Stage B non-rEF patient cohort underwent comprehensively analysis, with additional stratification into sub cohorts based on NYHA classification. Two approaches were used for stratification: (a) maximal oxygen consumption measured during cardiopulmonary exercise testing (NYHA CPET), and (b) symptomatic assessment using the Kansas City Cardiomyopathy Questionnaire (KCCQ) functional status score (NYHA KCCQ). The KCCQ functional score reflects the anamnestic assessment of NYHA class^25^. This approach was chosen because both maximal oxygen consumption and KCCQ scores are correlated with NYHA class and important clinical outcomes, such as mortality and hospitalization^25^. To identify potential similarities and differences, we performed stratification based on both assessments. Cut-off values of 21 ml/kg/min for maximal oxygen consumption and 80 for the KCCQ functional status score were applied to distinguish between NYHA Class I and Class II patients. These cut-off values were effectively established for stable HF patients in cohort assessments^26–29^, and we used the arithmetic mean difference between NYHA I and II for selecting cut-off values. Stratification by sex was also performed to identify specific characteristics in the correlation between KCCQ total score and exercise parameters.

### Procedures

#### Patient examinations and data collection

Patients had two examination days in the outpatient clinic. Day 1 consisted of collection of medical history by medical records, assessment of NYHA class^8^ via using the functional scale KCCQ (Kansas City Cardiomyopathy Questionnaire) and patient-reported outcome measures (KCCQ), a physical examination and blood sampling. The KCCQ provides an overall score ranging from 0 to 100 and evaluates health outcomes across three subscales: symptoms, clinical status, and functional capacity^25,30^. Out of medical history, we assessed comorbidities of special interest predisposing heart failure in our data set: ischemic heart diseases (clustered using ICD-10-WHO for: I25 coronary disease and ischemia, I21 heart attack and I70 coronary sclerosis), diabetes mellitus type II, hypertension and atrial fibrillation.

All procedures and diagnostics were done in accordance to our standardized operation procedures (SOP) which are based on guideline recommendations.

A lung function assessment was done to rule out moderate or severe chronical lung diseases. We reported clinical values for lung function assessments: forced vital capacity (FVC), one second vital capacity (FEV1) ratio (FEV1/FVC) and forced expiratory flow 25–75% of FVC (FEF 25–75).

Day 2 included the measurements of anthropometry and body composition (by bioimpedance analysis, Bodycorpus RX4004 SMT Medical), spirometry (Easy One Pro, NDD Medizintechnik AG, Switzerland), echocardiography (Vivid E95, GE Health Care) and a cardiopulmonary exercise test.

Cardio pulmonary exercise tests (CPET) were performed according to ergometry guidelines for cardiac patients^5^. Patients were examined on a semi-recumbent bicycle ergometer (ErgoSelect 10, Ergoline GmbH, Germany). After finishing of the patient preparation for the CPET, resting values were recorded over two minutes. The CPET began with an initial load of 30 watts and increased by 10 watts per minute. The test was performed at 60 to 75 revolutions per minute by the patients until voluntary exhaustion or occurrence of clinical reasons^31^. Blood pressure (Riva-Rocci-method)^32^ was measured at rest, every 3 min and at maximum load during the CPET and the first, third and fifth minute afterwards. This method is our standard measurement for clinical safety reasons. Beginning at rest, impedance cardiography (Manatec Physioflow, France) was recorded beat-by-beat and spiroergometry (Dynostics, Sicada GmbH, Germany) was recorded breath-by-breath until the fifth minute after the end of CPET. For further analysis, all measurements of continuous recorded values were averaged over 30nsecond intervals. The following calculations were used for physiological variables:

Arterio-venous oxygen difference (avDO_2_):

$$\:{\text{a}\text{v}\text{D}\text{O}}_{2}\:\left(\frac{\text{m}\text{l}}{\text{d}\text{l}}\right)=\frac{\text{o}\text{x}\text{y}\text{g}\text{e}\text{n}\:\text{c}\text{o}\text{n}\text{s}\text{u}\text{m}\text{p}\text{t}\text{i}\text{o}\text{n}\:\left(\frac{\text{m}\text{l}}{\text{m}\text{i}\text{n}}\right)}{\text{c}\text{a}\text{r}\text{d}\text{i}\text{a}\text{c}\:\text{o}\text{u}\text{t}\text{p}\text{u}\text{t}\:\left(\frac{\text{d}\text{l}}{\text{m}\text{i}\text{n}}\right)};$$

Left ventricular stroke work (SW):

$$\:\text{S}\text{W}\left(\text{J}\right)=\text{s}\text{t}\text{r}\text{o}\text{k}\text{e}\:\text{v}\text{o}\text{l}\text{u}\text{m}\text{e}\:\left(\text{m}\text{l}\right)\times\:\text{m}\text{e}\text{a}\text{n}\:\text{a}\text{r}\text{t}\text{e}\text{r}\text{i}\text{a}\text{l}\:\text{p}\text{r}\text{e}\text{s}\text{s}\text{u}\text{r}\text{e}\:\left(\text{m}\text{B}\text{a}\text{r}\right);$$

Left ventricular cardiac power output (CPO):

$$\:CPO\left(W\right)=\frac{\text{S}\text{W}\:\left(\text{J}\right)\:\times\:\:\text{h}\text{e}\text{a}\text{r}\text{t}\:\text{r}\text{a}\text{t}\text{e}\:\left(\text{b}\text{e}\text{a}\text{t}\text{s}\:\text{p}\text{e}\text{r}\:\text{m}\text{i}\text{n}\text{u}\text{t}\text{e}\right)}{60}.$$

Mean arterial pressure was calculated by heart rate-corrected calculation as described by Rogers and Oosthuyse^33^. The respiratory equivalent for oxygen (REVO_2_) was calculated:

$$\:REV{O}_{2}=\frac{\text{m}\text{a}\text{x}\text{i}\text{m}\text{u}\text{m}\:\text{m}\text{i}\text{n}\text{u}\text{t}\text{e}\:\text{v}\text{e}\text{n}\text{t}\text{i}\text{l}\text{a}\text{t}\text{i}\text{o}\text{n}\:\left(\frac{\text{l}}{\text{m}\text{i}\text{n}}\right)}{\text{o}\text{x}\text{y}\text{g}\text{e}\text{n}\:\text{c}\text{o}\text{n}\text{s}\text{u}\text{m}\text{p}\text{t}\text{i}\text{o}\text{n}\:\left(\frac{\text{l}}{\text{m}\text{i}\text{n}}\right)}\: .$$

We combined echocardiographic and thoracic impedance cardiographic assessment to incorporate the strengths of both methods^34^. Both, echocardiography and thorax impedance cardiography are able to measure the stroke volume by the ventricular outflow or the corresponding aortic flow^35^. The flow-based measuring method corresponding to thoracic impedance cardiography in echocardiographic assessments is the stroke volume measurement based on the systolic velocity time integral in the left ventricular outflow tract (VTI)^36^. Echocardiography is well-established as a valid and reliable method to determine the stroke volume at rest^37^. Stress echocardiography did not provide optimal conditions for CPET in terms of continuous measuring in patients and need a special positioning on the bicycle ergometer. Therefore we choose the, impedance cardiography as a reliable method for assessing continuous beat-by-beat hemodynamics during exercise^38,39^ and is also sensitive for changes in cardiac function in patients^39,40^ and therefore recommend as guideline-recommended monitoring assessment during CPET^5^. Calculating the stroke volume of impedance cardiography by relying on aortic flow and body surface area may overestimate the stroke volume in subjects with more body fat, as validation studies for exercise-related CO assessments included mostly healthy subjects^41,42^. Also, in our cohort of HF patients, chronical maladaptation of the heart and arterial blood vessels could affect the measuring accuracy. Therefore, we ran a cohort specific reliability analysis among the included patients to rule out systematic errors. Echocardiographic VTI-based stroke volume measurements for comparison methods were available in 28 patients.

### Statistics

Group differences were tested using a t-test for Gaussian distributions; otherwise, the Wilcoxon rank-sum test was applied. Homogeneity of variance was assessed with Levene’s test, and adjustments were applied if necessary. Correlations between physiological measures and the Kansas City Cardiomyopathy Questionnaire (KCCQ) scores were analyzed using Pearson’s correlation coefficient. Correlations were further examined for potential confounders, and the model was adjusted when significant influences were identified. Maximum oxygen consumption was normalized to body weight. Outliers were identified using the ROUT method (2% false discovery rate) for continuous variables used in group difference analyses and the k-nearest neighbor local outlier clustering method for correlation analyses.

Reliability analyses for stroke volume from echocardiography velocity-time integral (VTI) and impedance cardiography were assessed using the intra-class correlation coefficient (ICC) and a paired t-test. An a priori power calculation (G*Power, University of Düsseldorf, Germany) for the comparison of NYHA I and II heart failure (HF) patients indicated a power of 0.8 at a 0.05 significance level, assuming a clinically relevant mean difference of 10% and a standard deviation of 17%, requiring a sample size of 60 patients. We adopt a conservative mean difference of 10% for maximal oxygen consumption, based on results for NYHA I and II patients by previous studies that analyzed maximal oxygen consumption in NYHA I and II patients^5,29^. These studies reported a mean difference of 20% between NYHA I and II and a with a one-sided standard deviation of approximating 10%.

Values were presented as means and standard deviation (SD) or as numbers. Statistical analyses were performed using GraphPad Prism 8.0.2 and JASP 0.18.0.0. Graphics for the correlation matrix were created with Matplotlib 3.9.2 for Python. Correlations were considered as negligible below 0.1, weak from 0.1 to 0.39, moderate from 0.4 to 0.69, strong from 0.7 to 0.89 and very strong from 0.90 to 1.00^43^.

## Results

The results part consists of three sections. Patient characteristics and baseline parameters are presented in the first section and the results of the CPET in the second section. The third section provides the results for the correlation of the KCCQ score with physiological parameters.

### Patient characteristics and baseline parameters

Patient characteristics and resting values for the total cohort and for the sub cohorts of NYHA class I and class II, as well as gender specific sub cohorts are shown in Table 1. NYHA CPET class I and II patients differed in age, body fat and FEF 25–75%, but not in the prevalence of comorbidities. As expected, men and women differed in height, fat free mass and body fat percentage. NYHA KCCQ class I and II patients showed differences in in ejection fraction (EF) and the KCCQ scores. Lognormal distribution was found for NTproBNP and the Wilcoxon-Test was applied for testing group differences.

**Table 1:** Baseline characteristics.

	Total Cohort	NYHA I CPET	NYHA II CPET	NYHA I vs. II CPET (*p*-value)	NYHA I KCCQ	NYHA II KCCQ	NYHA I vs. II KCCQ (*p*-value)	Men	Women	Men vs. women (*p*-value)
n	62	32	30	n.s.	36	24		34	28	n.s.
Men/women (n)	34/28	19/13	15/15	n.s.	21/15	12/12	n.s.	34	28	< 0.001
Hypertension (n)	46	24	22	n.s.	29	17	n.s.			
Coronary heart disease (n)	21	10	11	n.s.	14	6	n.s.	15	6	n.s.
Diabetes mellitus II (n)	8	3	5	n.s.	4	4	n.s.	4	4	n.s.
Beta-blocker yes/no (n)	33/29	16/16	17/13	n.s.	24/12	9/13	n.s.	20	13	n.s.
Age (y)	68.40 (8.10)	65.81 (9.32)	71.17 (5.45)	= 0.004	68.47 (7.83)	67.75 (8.73)	n.s.	70.00 (6.02)	66.46 (9.83)	= 0.044
Height (cm)	172.7 (8.0)	173.4 (7.3)	171.9 (8.7)	n.s.	172.2 (7.9)	173.2 (8.5)	n.s.	177.9 (5.5)	166.4 (5.5)	< 0.001
Weight (kg)	76.10 (9.60)	75.44 (10.01)	76.80 (9.27)	n.s.	77.2 (9.3)	73.8 (10.1)	n.s.	81.32 (8.03)	69.75 (7.31)	< 0.001
Body mass index (kg/m^2^)	25.48 (2.31)	25.03 (2.47)	25.96 (2.05)	n.s.	26.2 (2.12)	24.5 (2.3)	= 0.003	25.70 (2.10)	25.22 (2.55)	n.s.
Fat free mass (kg)	55.46 (9.88)	56.03 (9.25)	54.83 (10.66)	n.s.	56.34 (10.32)	54.13 (9.46)	n.s.	62.73 (6.53)	46.31 (3.88)	< 0.001
Body fat (%)	27.36 (7.26)	25.78 (6.43)	29.10 (7.82)	= 0.038	27.89 (7.38)	26.61 (7.32)	n.s.	22.67 (4.86)	33.26 (5.18)	< 0.001
NTproBNP (ng/l)	242.7 (176.0)	220.8 (159.3)	266.1 (192.1)	n.s.	213.6 (99.8)	286.6 (250.6)	n.s.	259.4 (216.4)	222.4 (109.4)	n.s.
Ejection fraction (%)	59.16 (5.60)	58.94 (5.10)	59.40 (6.16)	n.s.	57.92 (5.40)	61.21 (5.62)	= 0.013	57.82 (5.12)	60.79 (5.81)	= 0.019
Heart rate (bpm)	69.21 (10.46)	70.25 (10.58)	68.10 (10.40)	n.s.	68.47 (8.49)	69.29 (12.729	n.s.	68.91 (10.4)	69.57 (10.75)	n.s.
SBP (mmHg)	143.10 (16.09)	140.80 (15.94)	145.70 (16.12)	n.s.	144.60 (15.85)	140 − 50 (16.43)	n.s.	145.10 (16.28)	140.70 (15.80)	n.s.
DBP (mmHg)	82.84 (8.29)	83.00 (8.04)	82.67 (8.69)	n.s.	83.83 (8.46)	81.79 (8.24)	n.s.	82.59 (9.22)	83.14 (7.16)	n.s.
Cardiac output (l/min)	7.09 (1.12)	7.07 (1.34)	7.12 (1.04)	n.s.	7.04 (1.20)	7.09 (1.15)	n.s.	7.245 (1.38)	6.91 (0.92)	n.s.
Stroke Volume (ml)	103.50 (17.68)	101.30 (18.11)	106.00 (17.19)	n.s.	103.50 (18.14)	104.0 (17.8)	n.s.	106.10 (18.94)	100.5 (15.83)	n.s.
FVC (l)	3.62 (0.86)	3.76 (0.74)	3.45 (0.96)	n.s.	3.61 (0.85)	3.55 (0.88)	n.s.	4.12 (0.71)	2.98 (0.55)	< 0.001
FEV1/FVC (%)	78.0 (7.62)	79.3 (7.21)	76.6 (7.93)	n.s.	77.94 (5.86)	77.92 (9.93)	n.s.	78.80 (8.80)	74.23 (15.80)	n.s.
FEF 75 − 25% (l)	2.59 (1.10)	2.84 (1.06)	2.31 (1.09)	0.028	2.56 (1.06)	2.56 (1.15)	n.s.	3.05 (1.08)	2.00 (0.76)	< 0.001
KCCQ summary score	78.3 (15.33)	79.63 (15.61)	76.78 (15.14)	n.s.	88.52 (6.93)	62.97 (10.989	< 0.001	78.25 (15.25)	78.4 (15.7)	n.s.
KCCQ symptoms score	80.2 (18.6)	80.67 (18.9)	79.72 (18.6)	n.s.	92.36 (6.98)	62.02 (15.35)	< 0.001	80.40 (18.61)	80.01 (18.92)	n.s.
KCCQ functional score	81.4 (15.3)	82.57 (15.26)	80.02 (15.5)	n.s.	91.37 (6.02)	65.85 (11.25)	< 0.001	81.85 (15.58)	80.81 (15.25)	n.s.
KCCQ clinical score	80.1 (15.8)	81.38 (16.18)	78.7 (15.6)	n.s.	90.57 (6.75)	64.47 (12.08)	< 0.001	79.95 (15.62)	80.36 (16.35)	n.s.

Entries are mean and standard deviation. Abbreviations: DBP: diastolic blood pressure, FEF: forced expiration flow, FVC: forced vital capacity, KCCQ: Kansas City Cardiac Questionnaire, NTproBNP: N-terminal pro–B-type natriuretic peptide, NYHA: New York Heart Association; SBP: systolic blood pressure.

### Reliability analyses between echocardiography and thoracic impedance cardiography

Sub analyses for reliability of echography and thoracic impedance cardiography revealed a good overall agreement by intra-class coefficient of *r* = 0.60 and no difference in mean stroke volume (echocardiography: 99.2 (SD: 23.5) ml vs. thoracic impedance cardiography: 99.4 (SD: 15.6) ml, *p*= 0.475). Our measured increase of 28% stroke volume (103.5 ml to 132.5 ml) during CPET at maximal exercise were in line with observed increase of stroke volumes in elderly of approximately 40%^44^. Based in the good overall agreement, stroke volumes assessments by thoracic impedance cardiography were considered as valid in our study cohort.

### Cardiopulmonary exercise test

The results of CPET of the total cohort and the sub cohorts for NYHA classes at maximum load are presented in Table 2 and gender specific data are presented in Table 3.

NYHA classes, classified via CPET, differed in peak power output, maximum oxygen uptake and carbon dioxide output, ventilation and respiratory equivalent for oxygen, heart rate and cardiac output, as well as avDO_2_.

**Table 2:** Results of CPET at maximum load for NYHA-Classes.

	Total	NYHA I CPET	NYHA II CPET	Significance NYH I vs. II CPET	NYHA I KCCQ	NYHA II KCCQ	Significance NYH I vs. II KCCQ
Power output (W)	107.4 (34.8)	119.1 (33.2)	91.1 (26.1)	< 0.001	113.3 (37.6)	99.6 (30.6)	n.s.
Relative oxygen consumption (ml/kg/min)	22.93 (6.1)	25.7 (4.3)	19.9 (3.8)	< 0.001	23.60 (5.68)	22.46 (4.67)	n.s.
Oxygen consumption (ml/min)	1753 (467)	1964 (456)	1523 (372)	< 0.001	1838 (504)	1654 (396)	n.s.
Carbon dioxide output (ml/min)	1665 (518)	1877 (540)	1455 (397)	< 0.001	1756 (586)	1560 (391)	n.s.
Respiratory exchange Ratio	0.95 (0.10)	0.94 (0.11)	0.95 (0.08)	n.s.	0.95 (0.10)	0.94 (0.95)	n.s.
Respiratory equivalent for oxygen	35.3 (6.6)	33.4 (6.2)	37.4 (6.6)	= 0.009	33.44 (5.37)	38.11 (7.33)	= 0.003
Ventilation (l/min)	60.6 (15.0)	65.6 (16.2)	54.3 (17.4)	= 0.001	60.8 (16.6)	61.2 (13.0)	n.s.
Heart rate (bpm)	129.8 (20.6)	132.4 (19.9)	120.8 (19.5)	= 0.004	129.2 (20.7)	131.4 (21.6)	n.s.
Stroke volume (ml)	132.5 (24.7)	131.3 (25.1)	130.0 (25.1)	n.s.	133.6 (23.1)	131.4 (28.2)	n.s.
Cardiac output (l/min)	17.2 (2.4)	17.4 (3.7)	15.8 (3.5)	= 0.002	17.3 (3.8)	17.1 (3.6)	n.s.
SBP (mmHg)	198.2 (24.1)	196.1 (27.6)	193.5 (22.2)	n.s.	198.8 (26.3)	194.8 (19.8)	n.s.
DBP (mmHg)	93.5 (11.0)	93.0 (9.3)	91.6 (11.6)	n.s.	94.4 (12.3)	91.3 (8.4)	n.s.
CPO (W)	5.26 (1.39)	5.34 (1.51)	4.65 (1.21)	0.001	5.34 (1.51)	5.13 (1.27)	n.s.
SW (Nm)	2.43 (0.53)	2.41 (0.59)	2.31 (0.49)	n.s.	2.47 (0.52)	2.36 (0.56)	n.s.
avDO_2_ (ml/dl	10.38 (1.94)	10.99 (1.86)	9.70 (1.84)	= 0.005	10.66 (1.76)	10.07 (2.21)	n.s.

Entries are mean and standard deviation. Abbreviations: avDO_2_: arterio-venous difference for oxygen; CPET: Cardio-pulmonary exercise test; CPO: cardiac power output, DBP: diastolic blood pressure, KCCQ: Kansas City Cardiomyopathy Questionnaire, NYHA: new York Heart Association; SBP systolic blood pressure, SW: stroke work.

Men and Women differed in all variables which are related to the body weight and in body weight independent variables avDO_2_ and SBP. No differences were found for ventilation parameters of respiratory exchange rate and the respiratory equivalent for oxygen.

**Table 3:** Results of CPET at maximum load for men and women.

	Men	Women	Significance (*p*-value)
Power output (W)	119.4 (35.2)	92.9 (28.8)	< 0.01
Relative oxygen consumption (ml/kg/min)	24.2 (5.4)	21.6 (4.8)	= 0.027
Oxygen consumption (ml/min)	1959	1502 (356)	< 0.001
Carbon dioxide output (ml/min)	1867 (525)	1419 (394)	< 0.001
Respiratory exchange ratio	0.95 (0.11)	0.94 (0.09)	n.s.
Respiratory equivalent for oxygen	35.3 (6.75)	35.4 (6.62)	n.s.
Ventilation (l/min)	67.6 (13.9)	52.2 (11.8)	< 0.001
Heart rate (BPM)	129.7 (22.9)	130.4 (16.7)	n.s.
Stroke volume (ml)	141.4 (21.9)	121.8 (24.0)	< 0.001
Cardiac output (l/min)	18.24 (3.4)	15.9 (3.6)	= 0.006
SBP (mmHg)	203.8 (25.6)	191.3 (20.6)	0.021
DBP (mmHg)	92.2 (12.2)	95.0 (9.2)	n.s.
CPO (W)	5.62 (1.34)	4.82 (1.34)	= 0.011
SW (Nm)	2.61 (0.48)	2.22 (0.50)	= 0.001
avDO_2_ (ml/dl)	10.84 (1.90)	9.81 (1.88)	= 0.020

Entries are mean and standard deviation. Abbreviations: avDO_2_: arterio-venous difference for oxygen, CPO: cardiac power output, DBP: diastolic blood pressure, SBP systolic blood pressure, SW: stroke work.

### KCCQ scores and correlation analysis with physiological parameters

**Fig. 2:**
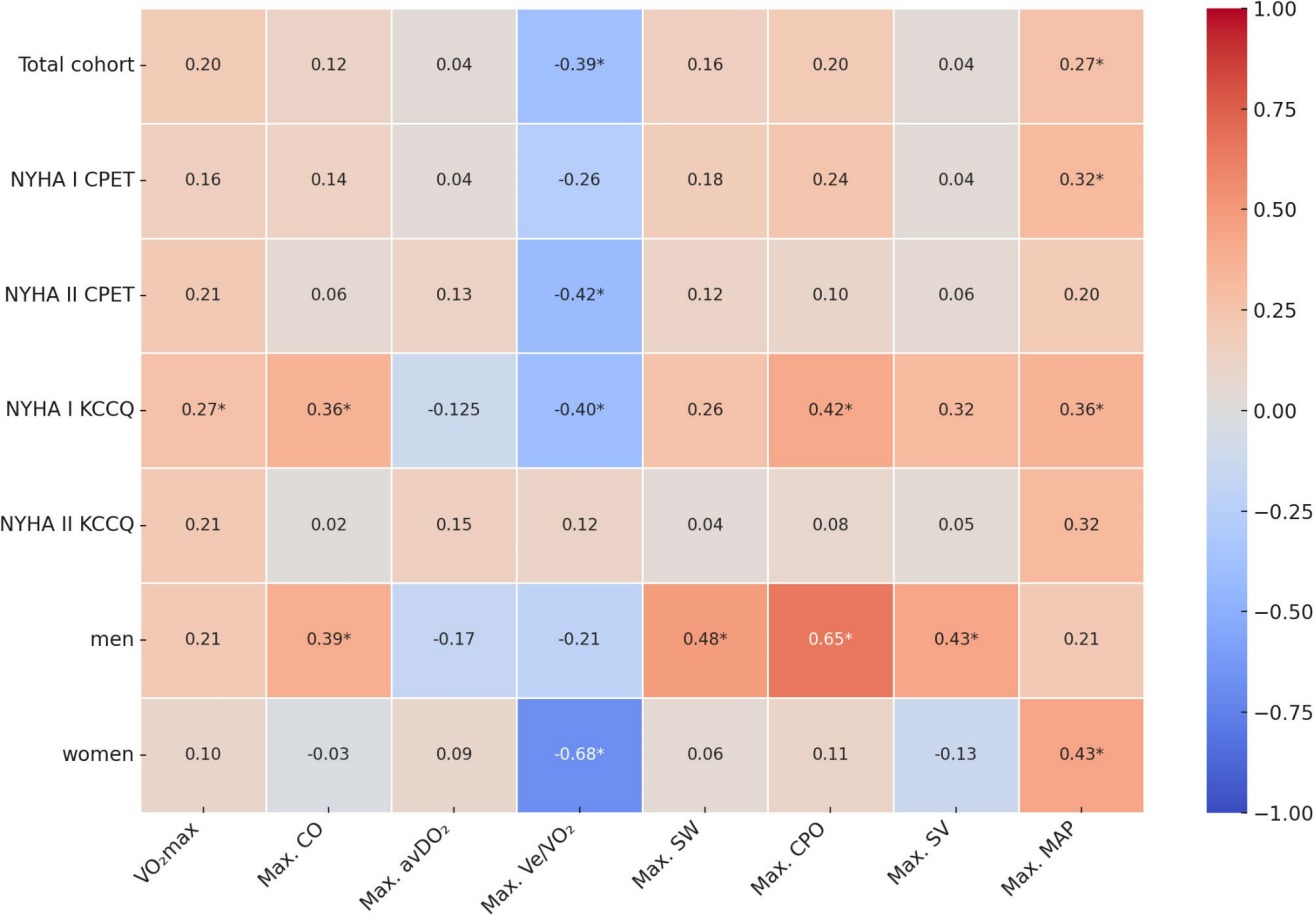
Correlation matrix for correlation coefficients of KCCQ-score in the total and sub cohorts. Significant correlations are marked with*.; Abbreviations: avDO_2_: arterio-venous difference for oxygen, CO: cardiac output, CPO: cardiac power output, CPET: cardiopulmonary exercise test, MAP: mean arterial pressure, NYHA: New York Heart Association, SV: stroke volume, SW: stroke work, Ve/VO_2_: respiratory equivalent for oxygen, VO_2_max: maximum oxygen consumption.

The correlations matrix (Fig. 2) illustrates the correlations between the KCCQ score and physiological parameters. Subscale correlation analysis revealed very strong correlations between the total KCCQ score and its subscales: the KCCQ symptom score (*r* = 0.95, *p* < 0.001), the KCCQ functional score (*r* = 0.97, *p* < 0.001), and the KCCQ clinical score (*r* = 0.99, *p* < 0.001). These correlations did not differ significantly from those observed for the total KCCQ score, confirming that the total score reliably represents all subscales. Consequently, we focused on the total KCCQ score in the correlation analysis with physiological parameters.

In the total cohort, the KCCQ score correlated significantly with the REVO₂ (*p* < 0.001) and mean MAP (*p* = 0.01). In the NYHA I CPET cohort, the KCCQ score correlated only with MAP (*p* = 0.04), whereas in the NYHA II CPET cohort, it was associated with REVO₂ (*p* = 0.015).

NYHA stratification by the KCCQ functional score revealed significant correlations within the NYHA I subgroup with the KCCQ total score: CO (*r* = 0.36, *p* = 0.016), REVO₂ (*r* = −0.40, *p* = 0.009), CPO (*r* = 0.42, *p* = 0.006), and MAP (*r* = 0.36, *p* = 0.016). In contrast, no significant correlations were observed between the KCCQ score and physiological parameters in the NYHA II KCCQ cohort.

**Fig. 3:**
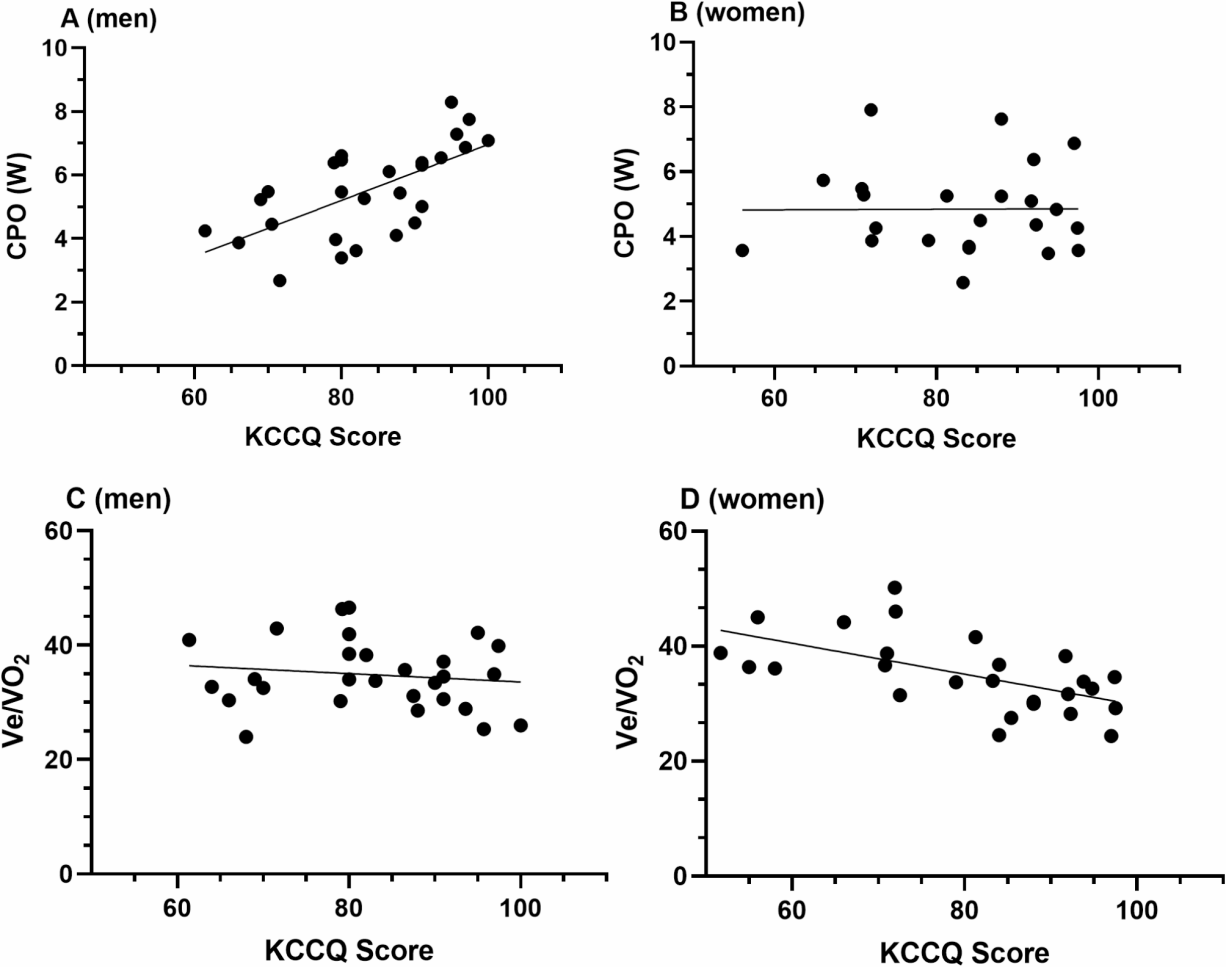
(**A**) CPO vs. KCCQ Score in men, (**B**) CPO vs. KCCQ Score in women. (**C**) Ve/VO_2_ vs. KCCQ Score in men, (**D**) Ve/VO_2_ vs. KCCQ Score in women.

The highest correlations were found within sex-specific subgroups. In men, higher KCCQ scores were strongly associated with increased CPO (*r* = 0.65, *p* < 0.001), while in women, the KCCQ score was significantly correlated with MAP (*r* = 0.43, *p* < 0.001), and inversely with REVO₂ (*r* = −0.68, *p* < 0.001). Figure 3 presents the correlations on subject level, demonstrating the different pattern between men and women for CPO and REVO_2_. In men were other significant correlations for cardiac markers with the KCCQ score: SW *r* = 0.48 (*p* = 0.004), max. SV *r* = 0.43 (*p* = 0.01), max. CO *r* = 0.39 (*p* = 0.017).

No correlations were observed between the KCCQ score and confounders age, height, weight, ejection fraction, lung function parameters, or BMI in any subgroup. Thus, these factors were not included as adjustments in the correlation analysis models. Dichotomous confounders such as beta-blocker use, diabetes mellitus type 2, and ischemic cardiac disease were also analyzed. A significant confounding effect was observed for beta-blocker use in women for REVO_2_. Inclusion of this confounder changed the correlation between REVO₂ and the KCCQ score from *r* = −0.58 to −0.68.

Outlier detection in the male subgroup identified six individuals in the CPO-KCCQ correlation. After excluding these individuals, a significant correlation between the KCCQ score and CPO was confirmed (*r* = 0.65, *p* < 0.001). There were no further statistical significant correlations.

## Discussion

This study explored in detail the relationship between NYHA classification, patient-reported outcomes (KCCQ scores), and objectified exercise capacity markers in Stage B HFpEF patients. By stratifying patients based on NYHA class via CPET or KCCQ functional score and sex, we identified significant patterns for differences in exercise capacity markers correlations to symptomatic outcomes.

### Resting values and patient characteristics

Our HFpEF cohort matched the reported characteristics of other heart failure cohorts, both symptomatically and in exercise capacity. Overall, the KCCQ score is consistent with other HF cohorts, which showed KCCQ scores of approximately 85 − 75 for NYHA class I and II patients^26–28^. Whereas mean values were typically found to be just above 80–85 for NYHA I and 75–80 for NYHA II. When NYHA was stratified by VO_2_max, our cohorts did not confirm this difference between NYHA I and II, neither in total nor in the sub scales of the KCCQ.

In our included patients, typical comorbidities predisposing for HFpEF were found (type 2 diabetes mellitus, hypertension or coronary diseases), consistent with other studies^45^. Incidences of coronary heart disease and diabetes mellitus type II are balanced between our NYHA groups, so prevalence of comorbidities was no major predictor for differences of NYHA classes in our results. The NYHA I and II cohorts differed in age, percentage of body fat and FEF 25–75%. Our NYHA cohort corresponds to a typical age range for heart failure patients under risk in Germany^45^. Within the total cohort, we found no significant correlation for age and KCCQ score or relative maximum oxygen consumption. Therefore, we did not adjust our results for age in our analysis. As Cleland et al. found a prevalence for 65% of men in heart failure patients, our cohort consist of a slightly lower 55% proportion of men^46^, which could be explained by the systematically screening of risk patients in an outpatient setting, whereas HF cohorts typically include symptomatic patients with cardiovascular diseases, which are more common in men. Further gender specific analyses in patient characteristics showed only differences which are related to known sex specific differences in height, weight or lean body mass.

### CPET and KCCQ-scores

Our total HFpEF cohort showed a major exercise limitation, expressed as reduced VO_2_max, in the skeletal muscle capacity (avDO_2_), which was consistent across all subcohorts. Both NYHA stratifications showed significant differences between NYHA I and II in REVO_2_, with higher ventilatory drive in NYHA II patients. When stratified by VO_2_max, the NYHA II group showed additional alterations in the CO and CPO (based on lower HR) and avDO_2_. Sex-specific differences, not related to a higher body weight in men, were lower relative VO_2_max, SBP and avDO_2_. The total KCCQ score showed the highest sex-specific correlations in sex-specific correlations: in men with CPO and in women with Ve/VO_2_ and MAP.

### Objective I: total cohort

Among our total HFpEF cohort, the CPET assessed oxygen uptakes at 22.9 ml/kg/min, reflecting HF typical levels. The relative maximum oxygen consumption in our NYHA class II patients of 19.9 ml/kg/min is in line with typical values of around 20 ml/kg/kg as well as the NYHA class I typically achieve values approximately of 25 ml/kg/min^6^. A major exercise limitation was the avDO_2_, which was reduced to almost half of the physiological upper limit^47^. The KCCQ score in the total cohort was only associated with the Ve/VO_2_ and the mean arterial pressure. Both physiological parameters are physiologically associated with the symptoms of dyspnea. The Ve/VO_2_describes a higher ventilator work for a given oxygen demand and workload and a dysregulation is therefore immediately noticeable for the patient as dyspnea under physical load. A higher MAP could be associated with a higher pulmonary arterial pressure^48^ or higher left ventricular filling pressures^49^, which also could lead to dyspnea. This underscores the importance of early blood pressure treatment and the need for early identification of patients at risk for heart failure^8^.

### Objective II: NYHA class I vs. NYHA class II patients

The difference between NYHA CPET class I and II patients in exercise performance is based on both higher cardiac output and higher muscular avDO_2_. With both groups achieving only intermediate maximal avDO_2_ values, skeletal muscle training status was the main exercise performance limiting factor in NYHA I as well as in NYHA II. As heart rate decreases naturally in age, and is partly compensated by higher stroke volumes, normally the avDO_2_remains stable^50^. Differences in NYHA groups in avDO_2_ are already known. Reddy et al. reported also an avDO_2_of 10.0 ml/dl in HFpEF patients, but did not separated for NYHA classes^51^. The peripheral limitation of HFpEF patient is well described in chronical and more progressed patient^16,52^. So, our patients are newly diagnosed HFpEF patients, and their avDO_2_ was already diminished on a level of chronical patients, which underlines the complexity in heart failure development by its syndromic frailty characteristic, even in the early diagnostic when patient were actively screened by physicians. This pattern could be confirmed in NYHA stratification by KCCQ functional score. In contrast to the NYHA classification via CPET the classification via KCCQ showed no differences in avDO_2_, CO or VO_2_max between the groups. Whereas a classification via CPET lead to a separation of patients via the total power output based on exercise stress reaction the KCCQ functional status includes the subjective perception of the patient. Therefore, the stratification based on the KCCQ functional score in NYHA I or II can be limited by a self-limiting behavior in the daily life and therefore the reduced exercise capacity will not be perceived by the patients. Also, in the beginning of heart failure patients did not recognize all symptoms and therefore the KCCQ functional score and clinical classification is not sensitive enough in newly diagnosed and actively screened HF cohorts. Changes in respiratory regulation are directly related to HF symptoms, therefore the Ve/VO_2_ may be more attributable to symptom scores.

Both NYHA stratifications showed an increased Ve/VO_2_, which is in line with recent findings^53^. Respiratoy equivalents for carbon dioxide > 35 are sensitive for symptomatic HFpEF patients which is in inline of our Ve/VO_2_ of 37.4 (NYHA II CPET) and 38.1 (NYHA II KCCQ).

By focusing on the cardiac capacity, we found a difference in the cardiac power output and cardiac output between the NYHA CPET classes, which are mainly caused by higher heart rates in NYHA I patients compared to NYHA II patients. Alterations in symptoms and functional capacity seems not only driven by the cardiac capacity and includes skeletal muscle and ventilatory alterations. The NYHA assessment by symptoms and exercise capacity assessment via CPET showed additive insights, but less regularity agreements in the analyzed outcomes.

### Objective III: sex-specific analysis

Men and women did not differ in the KCCQ scores in this study. Our demonstrated sex-specific differences in power output, absolute maximum oxygen consumption and cardiac parameters can be mostly attributed to the differences in height and weight between men and women.

The higher avDO_2_in men is mainly due to genetic differences is in line with demonstrated differences in elderly^54^. This 10% difference in avDO_2_ between men and women was the same size as the difference shown between NYHA class I and II patients and highlights the relevance of sex-specific reference values.

An additional main finding between men and women was found in the association of heart failure related symptoms to dedicated exercise capacity markers (Figs. 2 and 3). We firstly demonstrated a correlation in male HFpEF patients between KCCQ total score and the CPO and in female HFpEF with the Ve/VO_2_and the MAP. Sex-specific differences in HFpEF patients are known and often discussed^55–57^. Pulmonary circulation differences between men and women, especially in the pulmonary capillary wedge pressure, could account for differences in the genesis of symptoms in HFpEF patients^58^. The lower ventricular contractility reserve in women may explain the correlation of the MAP with the KCCQ^58^. It can be hypothesized that male HFpEF patients develop more pronounced symptoms as cardiac power output (CPO) declines and their higher contractility reserve diminishes over the course of HF progression.

Our findings the importance of sex-specific clinical exercise diagnostics in HFpEF patients and data analyses interpretations as relevant confounder.

## Conclusion

Our results showed that actively screened at-risk heart failure patients were similar to typical chronic heart failure patients in terms of symptoms and exercise capacity. This highlights the importance of early standardized diagnosis or screening for heart failure. Exercise testing is a valid tool to further assess overall work capacity and to differentiate the extent of cardiopulmonary and muscle-related symptom changes. Decreased muscle performance was a major limitation of exercise capacity. Improvements in exercise capacity as a therapeutic focus of exercise therapy in HF patients should be investigated in future studies. Exercise training may reduce this dysregulation by increasing avDO2, thereby reducing cardiac oxygen demand.

Notably, heart failure-related symptoms and patient outcomes showed the strongest correlation to exercise capacity markers in sex-specific sub cohorts. Functional classification by VO_2_max or KCCQ functional score showed similarities only for altered Ve/VO_2_ in NYHA II patients. Exercise markers assessed by CPET may reveal individual functional limitations. Our findings underscore the complexity of heart failure development, emphasizing the role of both physiological capacity assessments and patient-reported outcome measures. Depending on the stratification and sub cohort, we found association between heart failure symptoms and blood pressure, CPO, and REVO_2_. This underlines the need for early blood pressure treatment, exercise training, and gender-specific considerations in the management of heart failure patients at risk. Further research is necessary to reproduce our observations in larger real-world cohorts and to address targeted interventions to improve the quality of life and outcomes for patients at risk of heart failure.

## Limitations

Our study and interpretation had some limitations. The results of our study cannot be generalized to all patients with HFpEF due to our sample size and the limited sub cohort inclusion, for example, we could not include patients with arrhythmias such as atrial fibrillation in our cohort. Cohorts with larger numbers of patients may reveal more confounders and correlations in the association between PROMs and exercise variables, and thus provide further clinical insights. The impact of our findings on diseases progression and health care burden remains unclear as we did no follow-up assessments. The staging of HF patients is based on the chronic process of HF progression and my not be ideal for our actively screened cohort. We used CPET on a bicycle ergometer for physiological assessments, but these results should be extrapolated with caution to other types of exercise and activities.

## Data Availability

The datasets generated during and/or analyzed during the current study are not publicly available due to data protection for pseudonymized data but are partially available for aggregated data from the corresponding author on reasonable request.

## Abbreviations

AHA: American Heart Association
avDO_2_: Arterio-venous oxygen difference
BNP: Brain natriuretic peptide
BPM: Beats per minute
CO: Cardiac output
CPET: Cardio-pulmonary exercise test
CPO: Cardiac power output
DBP: Diastolic blood pressure
ESC: European Society of Cardiology
FVC: Forced vital capacity
FEV1: Forced expiratory volume for one second
HF: Heart failure
HFpEF: Heart failure with preserved ejection fraction
HFrEF: Heart failure with reduced ejection fraction
ICD-10: International classification of diseases, 10th revision
KCCQ: Kansas City cardiomyopathy questionnaire
MAP: Mean arterial pressure
NTproBNP: N-terminal pro brain natriuretic peptid
NYHA: New York Heart Association
Non-rEF: Non-reduced ejection fraction
Pmax: Maximum power output
SBP: Systolic blood pressure
SW: Stroke work
SPO: Stroke power output
Ve: Ventilation per minute
Ve/VO_2_: Respiratory equivalent for oxygen
VO_2_: Oxygen consumption
VO_2_max: Maximum oxygen consumption
VTI: Velocity time integral
WHO: World Health Organisation
